# MEG Evidence for Dynamic Amygdala Modulations by Gaze and Facial Emotions

**DOI:** 10.1371/journal.pone.0074145

**Published:** 2013-09-10

**Authors:** Thibaud Dumas, Stéphanie Dubal, Yohan Attal, Marie Chupin, Roland Jouvent, Shasha Morel, Nathalie George

**Affiliations:** 1 CNRS, UMR 7225, CRICM, Paris, France; 2 Inserm, U 975, Paris, France; 3 Université Pierre et Marie Curie-Paris 6, Centre de Recherche de l′Institut du Cerveau et de la Moelle Epinière (CRICM), UMR_S 975, and Centre MEG-CENIR, Paris, France; 4 CNRS, USR 3246, Centre Emotion, Hôpital Pitié-Salpêtrière, Paris, France; 5 CNRS, UMR 7295, CeRCA, Université François-Rabelais, Tours, France; University of Florida, United States of America

## Abstract

**Background:**

Amygdala is a key brain region for face perception. While the role of amygdala in the perception of facial emotion and gaze has been extensively highlighted with fMRI, the unfolding in time of amydgala responses to emotional versus neutral faces with different gaze directions is scarcely known.

**Methodology/Principal Findings:**

Here we addressed this question in healthy subjects using MEG combined with an original source imaging method based on individual amygdala volume segmentation and the localization of sources in the amygdala volume. We found an early peak of amygdala activity that was enhanced for fearful relative to neutral faces between 130 and 170 ms. The effect of emotion was again significant in a later time range (310–350 ms). Moreover, the amygdala response was greater for direct relative averted gaze between 190 and 350 ms, and this effect was selective of fearful faces in the right amygdala.

**Conclusion:**

Altogether, our results show that the amygdala is involved in the processing and integration of emotion and gaze cues from faces in different time ranges, thus underlining its role in multiple stages of face perception.

## Introduction

Over the past decades, the amygdala has been highlighted as a key structure in the perception of emotional and social stimuli, such as faces [1], [2]. The implication of the amygdala in socio-emotional processes was first illustrated with the description of the Klüver-Bucy syndrome in monkeys, which was characterized by profoundly abnormal emotional and social behavior following bilateral resection of the temporal lobe [3]. The description of patients with bilateral amygdala lesions then led to the hypothesis of a selective involvement of amygdala in the perception of fear [4], [5]. This view has evolved over the years to support the proposal of the amygdala as a key structure in the appraisal of stimulus relevance [6]–[8]. Accordingly, the human amygdala is particularly sensitive to faces that are highly relevant stimuli (even neutral faces; e.g. [9], [10]), and it has been shown to be sensitive to various facial signals including facial expressions and eye gaze. More precisely, the amygdala was found to be activated by faces conveying positive as well as negative emotions [11], [12]. It was also demonstrated to be responsive to seen gaze direction, with enhanced activation for neutral faces with direct relative to averted gaze in humans [13], [14], and to be involved in the attention orienting induced by gaze [15]. Moreover, the amygdala has been implicated in the integration of gaze and emotional expression cues from faces [16]–[20] (see also [21] for a recent discussion on the combined influence of gaze direction and emotion). However, while amygdala involvement in the processing of emotional expression and gaze cues from faces is well established, little is known about the dynamics of the neuronal responses in the human amygdala.

Much of what is known about the involvement of the amygdala in the perception of faces comes from fMRI and PET studies that do not allow unraveling the temporal dynamics of neuronal responses (see [22] for review). Direct intracerebral recordings of electroencephalographic (EEG) signals within the amygdala of epileptic patients have provided some insights onto the time course of amygdala responses to faces. Krolak-Salmon and coll. [23] found an increase of amygdala activity specific of fearful relative to neutral, disgusted, and happy faces between 200 and 800 ms. This effect was observed only when the task involved explicit processing of the emotional expression. In the same line, Meletti and coll. [24] showed amygdala responses selective to the eye region of seen faces between 200 and 400 ms; these responses were increased for fearful relative to neutral and happy expressions in a task where subjects were requested to pay attention to the seen emotion. By contrast, Pourtois and coll. [25] found differentiated amygdala responses to fearful and neutral faces between 140 and 290 ms, which were independent of the attention paid to the faces. Furthermore, Sato and coll. [26], [27] revealed an early increase (∼135 ms) of amygdala oscillatory activity in the gamma range (30–60 Hz) for fearful relative to neutral faces, as well as a later (∼200 ms) response to eye gaze in the same frequency range.

The development of electromagnetic brain imaging methods that allow source localization from non invasive electro- and magneto-encephalographic (EEG/MEG) scalp recordings has offered a new tool for the study of the temporal dynamics of brain responses in healthy subjects [28]. In recent years, an increasing number of studies have used these methods to investigate amygdala responses from MEG recordings in the context of emotion processing. For example, Cornwell and coll. [29] showed amygdala responses to emotional face matching vs. shape matching culminating between 136 and 188 ms after the face onset. In a series of studies using facial emotion recognition and face-versus-object categorization tasks, Streit and coll. [30], [31] and Liu and coll. [32] reported amygdala responses associated with facial emotion recognition between 100 and 220 ms. Another group reported responses to fearful versus neutral faces presented laterally between 80 and 160 ms [33], [34]. In addition, a few studies reported very early amygdala activation to threat-related faces, with activity starting from about 20 ms [35]–[37].

Although there are now more than 10 published studies in peer-reviewed high-quality journals that brought evidence for the localization of amygdala activity from MEG signals, there are still some debates about the possibility of observing amygdala responses with electromagnetic brain imaging methods. From this perspective, it is interesting to remind that the cortical grey matter is hypothesized to be the principal origin of the magnetic signal because of its organization in macrocolumns of pyramidal cells and its relatively small distance from the MEG sensors [38]. Nevertheless, the activity of the amygdala may also be detected due to its functional and histological properties. As a heterogenic structure, the amygdala is composed of distinct nuclei which differ from each other in their connectivity pattern and functional roles as well as in their histological properties. Among these nuclei, the basolateral nucleus, which is the major communication node between the neocortex and the amygdala, represents half of the total neurons in the human amygdaloid complex [39]. In rat amygdala, ninety-three percents of these neurons have been identified as pyramidal cells – the main sources of the MEG signal from the brain [40]. Although the pyramidal cells in the basolateral nucleus do not show a laminar organisation, it is well possible that the activation of subpopulations of these cells in response to relevant stimuli may produce a non-zero net current dipole source. In support of this view, electrophysiological recordings in the amygdala of non-human primates have isolated different groups of selectively responsive cells, including face-specific neurons as well as neurons selectively activated by threat-related stimuli [41]–[45] (see also [46]). Moreover, with an average volume of 44.5 mm^3^ and a composition of 12.2 millions of neurons [39], the human amygdala has a mean density of 272 millions of neurons/cm^3^. By contrast, the density of neurons in the neocortex is estimated to 44 millions of neurons/cm^3^ [47]. Thus, the amygdala is six times as dense as the neocortex. Accordingly, we have shown in a simulation study using a growing patch of activation in the amygdala that an activated volume of 0.2–0.3 cm^3^ was sufficient to generate a magnetic signal above the noise level of MEG sensors [48], [49]. Altogether, this may explain why, although the amygdala may be qualified as a deep brain structure, it may still contribute significantly to the magnetic signals recorded at the scalp surface and be detectable with MEG, as demonstrated in the above mentioned papers (see also [50], [51]).

Here, we wanted to further investigate the time course of amygdala responses to faces using MEG. We combined a distributed Minimum Norm Estimate method for source reconstruction with an anatomical segmentation method developed by Chupin and coll. [52]. This segmentation method allowed us to localize the amygdala in each individual subject and to take into account a volumic grid of dipoles placed within this structure in addition to the sources distributed over the cortical surface. We examined amygdala responses to fearful and neutral faces with direct and averted gaze. Adolphs [53] has proposed that the same brain structure may participate in different components of processing at different points in time. Thus we wanted to investigate whether the amygdala may be sensitive to emotional expression and eye gaze cues in different time intervals. Our hypothesis was that there may be early amygdala responses to the faces, which should differentiate fearful and neutral faces [25], [26], [32], [34]. Following Sato and coll. [27] findings, the effect of seen gaze direction was expected to emerge later. An interesting question was that of the integration of gaze and expression cues: Current anatomo-functional models of face processing postulate relatively late stages of integration of these facial cues (e.g.; [54], [55]) but it remains an open empirical question. In addition, we measured the anxiety level of the participants in order to investigate if anxiety might modulate amygdala responsiveness to emotion and gaze cues, as was previously found in fMRI studies [56]–[60] (for reviews, see [61], [62]). A recent fMRI study has shown a correlation between state anxiety and the activity in the amygdala and extended amygdala regions related to the integration of gaze and facial expression [63]. Thus, we wanted to test if amygdala activity in response to fearful and neutral faces with direct and averted gaze depended on the participants’ anxiety level, and if anxiety may modulate amygdala activity from the early stages of stimulus processing and/or over sustained time intervals.

## Materials and Methods

### Participants

Fifteen healthy volunteers took part in this study (11 female, mean age 26.1±3.3 years). All were right-handed, had normal or corrected to normal vision and declared no history of neurological or psychiatric disorders. They provided written informed consent and were paid for their participation. All procedures were approved by the local ethics committee (“Comité de protection des personnes Ile-de-France VI”, CPP Idf VI).

Participants completed the Spielberger State-Trait Anxiety Inventory (STAI) [64]. Participants’ state anxiety scores ranged from 20 to 38 (mean = 26, SD = 5.20). These scores are similar to published norms for this age group.

### Stimuli

Faces from 16 different individuals were selected from the Karolinska Directed Emotional Face database [65] under their fearful and neutral frontal view versions. An averted gaze version was obtained for each of these stimuli, by manually modifying the eye positions of the faces, using Adobe Photoshop 7.0.1. This resulted in four experimental conditions in a 2×2-factorial design with emotion (fearful or neutral) and gaze direction (direct or averted) as orthogonal factors. All pictures were set to gray level, resized and cropped to an oval shape. Global luminance and contrast of each stimulus (measured inside the oval shape) were modified using Adobe Photoshop 7.0.1 in order to ensure that there was no significant difference between the experimental conditions (mean grey levels = 60.7±10.8/60.8±10.8/60.3±10.8/60.4±10.7 for neutral faces with direct and averted gaze and fearful faces with direct and averted gaze, respectively; global contrast = 30.8±2.6/30.7±2.6/30.1±2.5/30.1±2.5 for the same conditions). Faces subtended a visual angle of about 6 degrees (vertically) and 3.5 degrees (horizontally).

### Procedure

Participants were comfortably seated inside an electromagnetically shielded MEG room in front of a translucent screen placed at a viewing distance of 82 cm. Stimuli were back projected onto the screen through a video projector placed outside of the room and two mirrors inside the MEG room. Stimulus presentation was controlled by a computer equipped with OmniStim, a home-made software, and connected to the MEG data acquisition computer through the parallel port.

**Figure 1:**
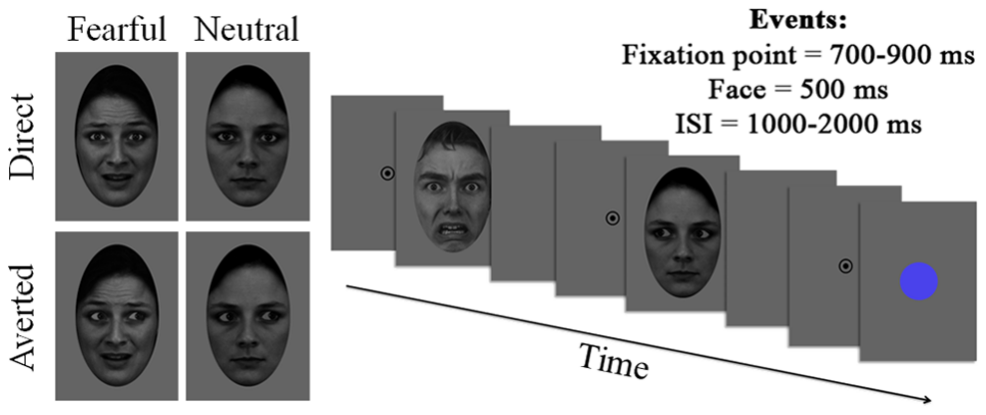
Experimental conditions and trial timeline. On the left: Example stimuli for the four categories of faces used (fearful and neutral faces with direct and averted gaze). On the right: Illustration of the trial timeline. Each trial started with a fixation point (700–900 ms) followed by the presentation of a face (500 ms), and a blank screen (ISI: 1000–2000 ms) before the next trial started. The participant’s task was to press a button on the occurrence of occasional blue circle targets.

Each trial started with a central fixation point displayed for 700 to 900 ms. Then a face stimulus was displayed for 500 ms, followed by a blank screen presented for 1 to 2 seconds before the next trial started (Figure 1). There were six blocks of 64 trials (total = 384 trials). In each block, the 16 different faces were seen once under each of the 4 experimental conditions of gaze (direct/averted) and emotion (fearful/neutral). Eight to twelve target stimuli consisting in a centrally presented blue dot were added to each block for the purpose of the task. The subjects were instructed to press a button whenever they detected these target stimuli. All subjects performed the task with ceiling performance (mean number of target detected, across subjects = 57±1 over the total of 60 targets).

### MEG Recordings

The study took place at the MEG Center of the Centre de Neuro-Imagerie de Recherche (CENIR, CRICM – UPMC/Inserm/CNRS), Paris, France. Magnetoencephalographic signals were collected continuously on a whole-head MEG system with 151 axial gradiometers (Omega 151 CTF Systems, Port Coquitlam, British Columbia, Canada) at a sampling rate of 1250 Hz with a 200 Hz low-pass filter. Seventeen external reference gradiometers and magnetometers were used to apply a synthetic third-gradient to all MEG signals for ambient field correction. Three small coils were attached to reference landmarks on the participant (left and right pre-auricular points, plus nasion) in order to monitor head position and to provide co-registration with the anatomical MRI. Head position was recorded and controlled before each stimulus block. The recording also included the signal of a photodiode that detected the actual appearance of the stimuli on the screen within the MEG room. This allowed correcting for the delay introduced by the video projector (20 ms) and averaging event-related magnetic fields (ERFs) precisely time-locked on the actual onset of the face stimulus. Vertical and horizontal eye movements were monitored through bipolar Ag/AgCl leads placed above and below the subject’s dominant eye, and at the outer canthi of each eye, respectively.

### MRI Acquisition

Structural MRI scan was obtained on a Siemens 3T Trio TIM scanner operated in the CENIR, CRICM – UPMC/Inserm/CNRS, Paris, France (MPRAGE sequence, TR: 2300 ms, TE: 4.18 ms, FA: 9°, voxel size: 1×1×1 mm^3^, sagittal scans, 248×256 voxels/slice, 176 slices).

### Event-related Magnetic Fields (ERFs)

Trials contaminated by eye movements, blinks or muscular activity were rejected manually upon visual inspection of the MEG and EOG signals. The mean number of trials included in the ERF averages did not differ across conditions (mean number of trials±SEM averaged: 81.4±2.8, 80.8±3.1, 82.1±2.9 and 80.9±3.0 for fearful faces with direct gaze, fearful faces with averted gaze, neutral faces with direct gaze, and neutral faces with averted gaze respectively; all F(1,14)<1.5, all p>.2). Event-related magnetic fields (ERFs) were then averaged for each condition between −200 ms and +600 ms (0 ms = face onset). Finally, data were baseline corrected according to the 200 ms preceding face onset, and digitally low-pass filtered at 40 Hz.

### Source Localization

#### Forward problem

**Figure 2:**
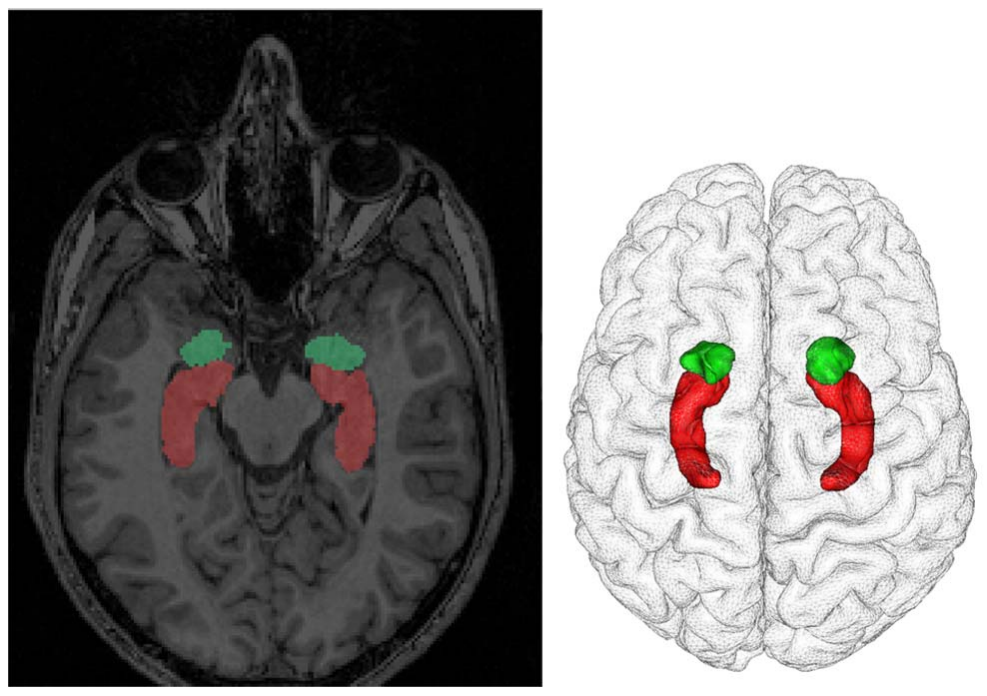
Illustration of the anatomical segmentation of the amygdala and the hippocampus from the individual T1 MRI scan of a typical subject. On the left: Amygdala (in green) and hippocampus (in red) segmentation masks obtained with the method of Chupin and coll. (2007) are visualized on a horizontal view of the participant’s anatomical MRI. On the right: Top view of the tessellated surfaces of the amygdala (in green) and the hippocampus (in red) merged with the tessellated cortical surface (obtained with BrainVisa) from the same individual’s MRI scan.

For each subject, a tessellated envelope of the neocortex was obtained with The Anatomist/brainVISA free software solutions (http://brainvisa.info). Moreover, the amygdala was segmented from each individual MRI with the recently developed method of [52]. This method is based on a competitive region-growing approach and was operated in the brainVISA environment. It provides tessellated surfaces of the amygdala and hippocampus. Thus, we obtained three tessellated surfaces that were used to distribute the elementary equivalent current dipoles (ECD) of our source imaging model (Figure 2). ECDs were evenly distributed perpendicularly to the surfaces of the neocortex and of the hippocampus, in order to model the macrocolumns of pyramidal cells in these structures. Considering the heterogeneous composition of the amygdala in terms of nuclei and histological aspect, we chose to transform the segmented amygdala surface into a volumic grid. Orthogonal trihedral ECDs were then placed at each node of this volumic grid for each subject [50]. The average number of sources distributed in the amygdala model is summarized in Table 1. Overlapping sphere method was then used to compute the head model for each subject with Brainstorm toolbox [66], using the individual head mesh and sensor locations [67]. Brainstorm toolbox is documented and freely available for download online under the GNU general public license (http://neuroimage.usc.edu/brainstorm). The gain matrices were then obtained for each structure (neocortex, amygdala, and hippocampus) and concatenated.

**Table 1:** Mean number of sources (±SEM) distributed in the amygdala volume and over the cortical surface across subjects.

Structures	Number of sources	
	Right hemisphere	Left hemisphere
Amygdala	386±15	396±18
Neocortex	12448±288	12484±268

#### Inverse problem

For each subject and each condition, the amplitude of the activation of each ECD of the neocortex, amygdala, and hippocampus was estimated at every time point by the ‘Deep Brain Activity’ (DBA) distributed source imaging model [50] that is based on weighted Minimum Norm Estimation [68] computed with the default values of Brainstorm (weighting factor = 0.4; Tikhonov parameter = 10% of the maximum singular value of the lead field).

For the amygdala, the norm of the vector resulting from the trihedral sources was computed at each node of the volumic grid. The mean time course of amygdala activity was then obtained by averaging all vector norms in the amygdala volume, separately for the left and right amygdala, for each subject and each condition.

For the neocortex, the time-resolved individual cortical maps were projected onto the default anatomy of Brainstorm toolbox that consists in the segmented cortical surface (15000 vertices) of the MNI/Colin27 brain [69]. These data were transformed into z-score with respect to the mean and standard deviation of dipole current amplitude during the baseline period and grand averaged across subjects for the purpose of region-of-interest definition.

### Data Measurements and Statistical Analyses

We measured the mean amplitude of the amygdala activity in two time ranges selected on the basis of the peaks of activity obtained across subjects: i) between 130 and 170 ms, to encompass the early prominent peak of amygdala response across subjects, and ii) in four consecutive 40-ms time windows between 190 and 350 ms, to encompass the secondary peaks of amygdala observed and that resulted in a sustained response from about 200 ms across subjects. These data were analyzed using analyses of covariance (ANCOVA) with emotional expression (fearful/neutral), gaze (direct/averted), and hemisphere (left/right) as within-subject factors, and the participant’s anxiety score as a covariate. The window of measurement (190–230 ms/230–270 ms/270–310 ms/310–350 ms) was introduced as an additional within-subject factor for the analysis of the mean amplitude of amygdala activity between 190 and 350 ms. Greenhouse-Geisser correction was applied for the comparisons of more than one degree of freedom; the Greenhouse–Geisser epsilon (ε_GG_) value for the adjustment of the degrees of freedom is then reported.

For the neocortex, two regions of interest (ROIs) – where a prominent peak activity was observed in the same time range as in the amygdala – were defined in the ventral and lateral occipito-temporal regions. These ROIs were centered on the maximum of activation observed on the grand averaged z-score normalized cortical maps between 130 and 170 ms. The raw (non-normalized) time series of current dipoles were then extracted from these ROIs (77 and 82 vertices respectively) for each subject and each condition, and the mean current amplitude was computed in each ROI in the same time windows as for the amygdala. These data were analysed using ANCOVAs in the same way as amygdala activity.

## Results

**Figure 3:**
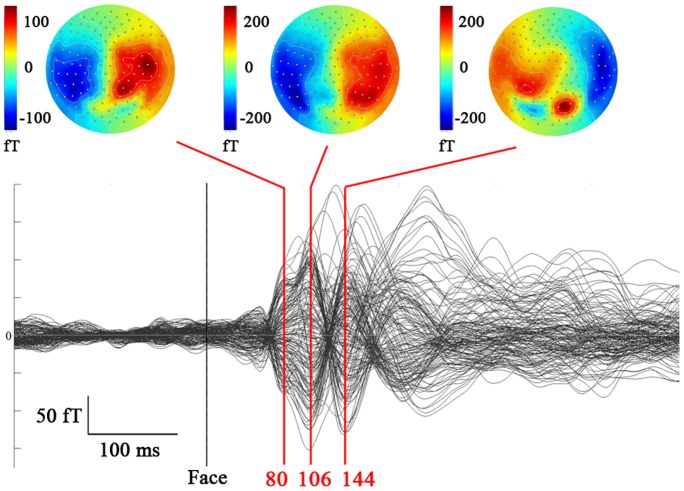
Event-related magnetic fields (ERFs) in response to faces. On the top: Maps (top-view of the head) of the ERFs at 80, 106 and 144 ms, averaged across all subjects and conditions. Below: Superimposed time courses of the ERFs over the 151 sensors, averaged across all subjects and conditions.

As can be seen on Figure 3, the grand mean of event-related magnetic fields (ERFs) at sensor level showed the classical succession of visual ERF components with a first peak at 80 ms post-stimulus onset followed by a prominent peak of magnetic signal at 106 ms (M100), and then the typical M170 pattern to faces – with a main flowing-in field over the right hemisphere and a main flowing-out field over the left hemisphere – that peaked here at 144 ms. We localized the sources of magnetic activity in each subject using individual amygdala volumes in addition to the cortical surface as our source imaging model in order to investigate amygdala responses to fearful and neutral faces with direct and averted gaze.

### Amygdala

**Figure 4:**
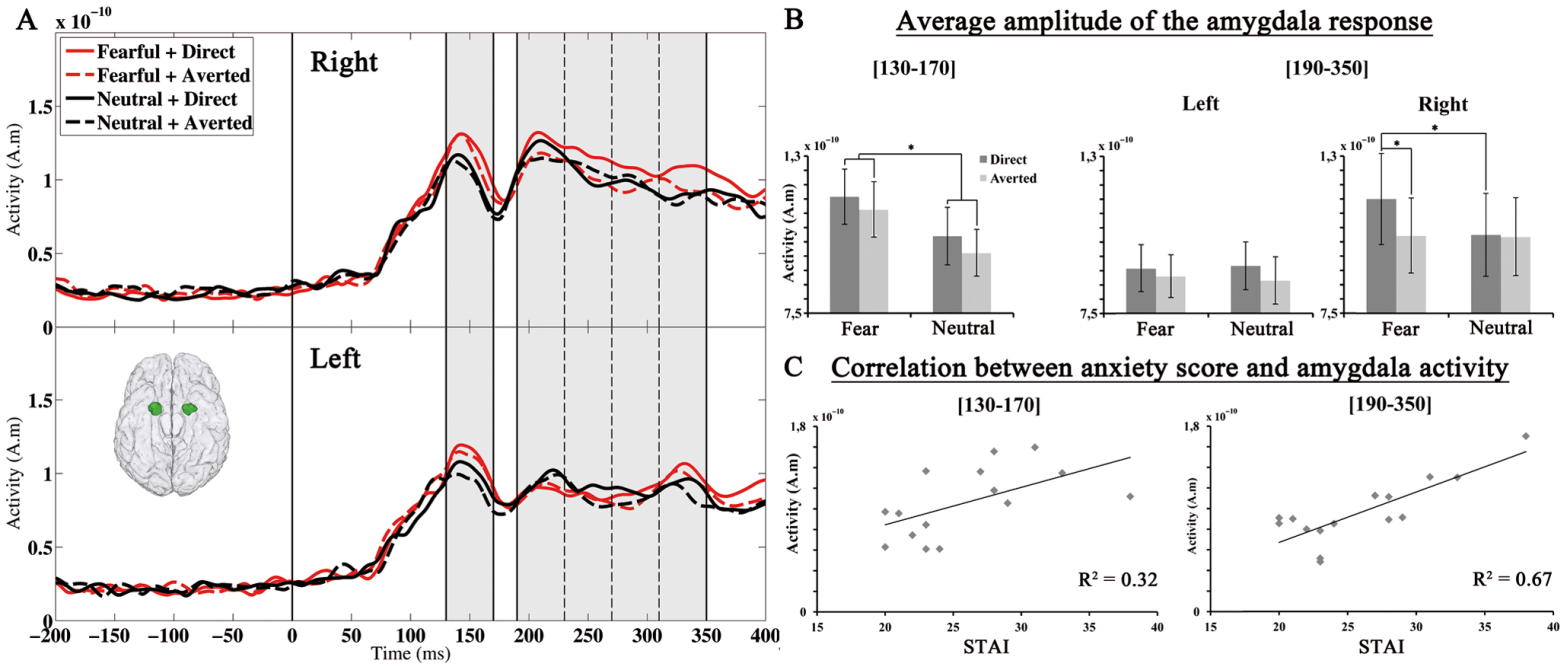
Amygdala responses to the faces. A) Time course of the right and left amygdala responses to the fearful (in red) and neutral (in black) faces with direct (plain line) and averted gaze (dashed line). The amygdala activity averaged across the 15 subjects is presented. The time windows where the mean amplitude of amygdala activity was measured are shaded in grey. B) Plots of the effect of emotion and gaze on amygdala activity between 130 and 170 ms and between 190 and 350 ms. On the left: A main effect of the emotion conveyed by the face was observed between 130 and 170 ms. On the middle and right: A main effect of gaze direction qualified by an interaction with emotion and hemisphere was observed between 190 and 350 ms. This reflected a significantly greater response to fearful faces with direct gaze than to fearful faces with averted gaze and to neutral faces with direct gaze in the right amygdala. On every plot, the error bars represent the standard errors of the means across subjects (SEM). C) Correlation between the amygdala activity and the participants’ anxiety score (STAI). This correlation was observed in both time ranges of amygdala activity measurement (130–170 ms and 190–350 ms).

The activity extracted from amygdala sources showed a sharp increase from about 70 to 80 ms post-stimulus onset, reaching an early maximum between 100 and 170 ms in every subject, which resulted in an averaged prominent peak of activity at ∼140 ms (Figure 4A). This was followed by secondary peaks of activity reflected in an across-subjects averaged sustained response from about 200 ms. We performed mean amplitude measurements of these amygdala responses to the faces.

First, we analyzed the mean amplitude of amygdala activity between 130 and 170 ms in order to encompass the major part of the early prominent peak response across subjects. The ANCOVA with emotional expression (fearful vs. neutral), gaze direction (direct vs. averted), and hemisphere (left vs. right) as within-subject factors, and anxiety level as a continuous covariate showed a significant main effect of emotion (F(1, 13) = 29.14, P = .0001); this revealed greater mean amplitude of amygdala activity in response to fearful than to neutral faces between 130 and 170 ms (Figure 4B). There was also a main effect of the anxiety covariate (F(1, 13) = 6.08, p<.05), showing that amygdala activity increased with the participant's anxiety level (Figure 4C). The ANCOVA did not reveal any significant interaction between emotion and anxiety level (F<1), suggesting independent effects of these variables. In other words, anxiety level modulated the mean amplitude of amygdala activity, but it had no significant influence on the effect of emotion, between 130 and 170 ms. There was not any other significant main effect or interaction.

Second, in order to get some insight into the temporal unfolding of the following sustained activity, we measured the mean amplitude of amygdala activity in 4 consecutive 40-ms time windows between 190 and 350 ms (190–230 ms/230–270 ms/270–310 ms/310–350 ms). The ANCOVA with emotional expression, gaze direction, hemisphere, and time window as within-subject factors, and anxiety level as a continuous covariate, did not show any significant effect of emotion. Yet there was an interaction between emotion and time window (F(3,39) = 4.23, ε_GG_ = 0.64_,_ p<.05), reflecting an increase of the mean amplitude of amygdala activity in response to fearful relative to neutral faces between 310 and 350 ms only (F(1, 13) = 10.83, p<.01). Moreover, there was a significant main effect of gaze direction (F(1, 13) = 7.59, p<.02): the mean amplitude of amygdala activity between 190 and 350 ms was greater for faces with direct gaze than for faces with averted gaze. In addition, the three-way interaction between gaze direction, emotion, and hemisphere was significant (F(1, 13) = 5.11, p<.05). Planned comparisons revealed that there was an increased amygdala response to fearful faces with direct relative to averted gaze in the right amygdala (F(1, 13) = 5.44, p<.05). No such effect of gaze direction was observed for neutral faces (F<1). The three-way interaction also reflected that right amygdala activity between 190 and 350 ms was greater for fearful than neutral faces only when the faces were seen under direct gaze (F(1, 13) = 7.88, p<.02; F<1 for the effect of emotion under averted gaze). By contrast, in the left amygdala, there was only a trend to a main effect of gaze (F(1, 13) = 4.11, P = .06), revealing globally greater amplitude of amygdala response for faces with direct gaze compared to faces with averted gaze. Finally, the effect of anxiety level was significant (F(1, 13) = 26.72, p<.0005), indicating that the mean amplitude of amygdala activity between 190 and 350 ms covaried with the participant’s anxiety level. There was no other significant effect or interaction.

### Cortical Sources

**Figure 5:**
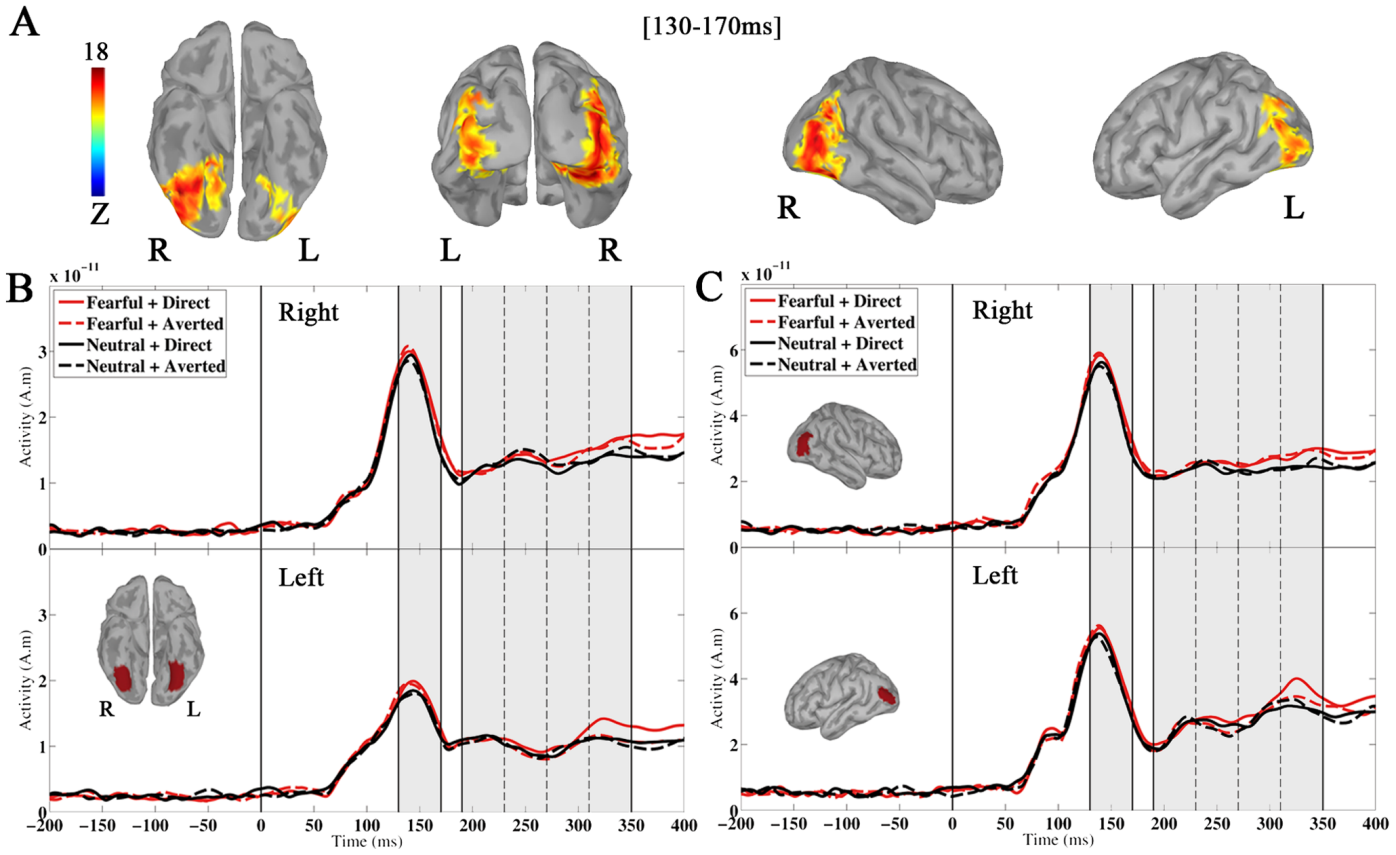
Cortical sources of activity. A) Mean cortical current maps between 130 and 170 ms. The colour-coded activity of cortical dipole sources (in z-score units), averaged across all subjects and conditions, is superimposed on the ventral, back, right and left lateral views of an inflated template brain. Only sources with amplitude above 60% of the scale maximum activity are displayed. B) Time course of cortical source activity in fusiform regions under each experimental condition. The cortical source activity averaged across all subjects over the right and left fusiform clusters respectively (displayed in red on a ventral view of the brain, in a small inset) is presented. C) Time course of cortical source activity in lateral occipital regions under each experimental condition. The cortical source activity averaged across all subjects over the right and left lateral occipital clusters respectively (displayed in red on lateral views of the template brain, in small insets) is presented. The time windows where the mean amplitude of cortical source activity was measured are shaded in grey.

We examined the cortical sources of magnetic activity concurrent with amygdala peak responses. Prominent cortical activities were observed between 130 and 170 ms in the bilateral fusiform regions, predominantly in the right hemisphere, extending into the lateral occipital regions (Figure 5A). No other cortical sources of magnetic activity were observed between 190 and 350 ms. We defined four source clusters encompassing the early peak of activity observed in the right and left fusiform and lateral occipital regions respectively (Figure 5B and C). We performed mean amplitude analyses of the activity from each of these clusters in the same time ranges as for the amygdala.

As for the fusiform source clusters, the ANCOVA performed on the mean amplitude of fusiform responses between 130 and 170 ms showed a main effect of emotion (F(1, 13) = 22,72, p<.0005) and a main effect of anxiety level (F(1, 13) = 9.39, p<.01). The mean amplitude of fusiform responses was greater for fearful than for neutral faces and it increased with the participant's anxiety level. There was also a significant effect of hemisphere (F(1, 13) = 5.87, p<.05) that reflected greater activity in the right than in the left fusiform region. The ANCOVA did not reveal any other significant main effect or interaction.

Furthermore, the analysis of the mean amplitude measurement of fusiform activity in 4 consecutive 40-ms time windows between 190 and 350 ms showed a significant interaction between emotion and time window (F(3,39) = 5.68, ε_GG_ = 0.76, p<.005), indicative of an enhanced fusiform activity for fearful compared to neutral faces between 310 and 350 ms only (F(1, 13) = 10.83, p<.01). There was not any significant effect of gaze (F<1). The interaction between emotion and gaze was significant (F(1, 13) = 4.99, p<.05) revealing an effect of emotion on fusiform activity (in the form of enhanced response to fearful faces) under direct gaze only (F(1, 13) = 6.44, p<.05). No other effect or interaction reached significance.

As for the lateral occipital source clusters, the ANCOVA performed on the mean amplitude of lateral occipital responses between 130 and 170 ms showed a main effect of emotion (F(1, 13) = 7.89, p<.05) and a main effect of anxiety level (F(1, 13) = 7.52, p<.05). The mean amplitude of lateral occipital responses was greater for fearful than for neutral faces and it increased with the participant’s anxiety level. No other effect or interaction reached significance.

The ANCOVA performed on the mean amplitude of lateral occipital activity between 190 and 350 ms showed a significant interaction between emotion and time window that reflected greater lateral occipital activity for fearful than neutral faces in the 310–350 ms time window only (F(1, 13) = 18.80, p<.001). There was also a main effect of anxiety level (F(1, 13) = 4.70, p<.05) that showed that the mean amplitude of lateral occipital activity between 190 and 350 ms covaried with anxiety level. No other significant effect or interaction was found.

## Discussion

The present study aimed at investigating the temporal dynamics of amygdala activity in response to faces with different emotional expressions (fearful/neutral) and gaze directions (direct/averted). Our hypothesis was that amygdala may participate in multiple stages of face processing in different time windows, with effects of emotion and gaze in different time ranges. In addition, subject’s anxiety level was taken into account as a modulatory variable. We used MEG together with an original source estimation technique based on the anatomical segmentation of medial temporal lobe structures to localize the amygdala volume in single subjects. We found amygdala activation starting from about 80 ms after face onset and reaching a prominent peak of activity at about 140 ms. Emotion modulated amygdala activity in two time ranges: between 130 and 170 ms, and later between 310 and 350 ms. Gaze direction influenced amygdala activity in a different time-range (190–350 ms). In addition, there was a sustained modulation of amygdala activity by anxiety level.

The first effect of emotion was observed between 130 and 170 ms with larger amplitude of amygdala activity for fearful compared to neutral faces. This is consistent with the recent papers that proposed other methods to estimate amygdala sources from MEG signals and showed emotional modulation of amygdala activity in similar time-ranges [29], [31], [32], [35], [37], [70], [71]. Furthermore, the timing of our first peak activation in the amygdala coincides with the early peak of amygdala activity modulated by emotional expression as reported in a recent intracranial ERP study [25], [26]. This early peak of amygdala activity may reflect an initial, rapid stage of face appraisal and emotion detection. Indeed, the subjects of our study were engaged in a simple task of detection of an occasional blue circle target. Thus amygdala activity in response to the faces may be considered as reflecting the automatic processes elicited when viewing faces with neutral and fearful expressions. This view is in line with that of Pourtois and coll. [25] and Sato and coll. [26]. It extends these previous studies by bringing converging evidence in healthy subjects using non-invasive source localization method from MEG signals. Our result of early emotional modulation of amygdala activity during passive fixation of faces is also consistent with the appraisal theory of amygdala function: Emotions conveyed by faces constitute particularly relevant stimuli that may be automatically and rapidly appraised, and amygdala has been proposed as a key relevance appraisal structure [72].

Most interestingly, we also found emotional modulation of amygdala activity in a late time window (310–350 ms). This late modulation is likely to reflect a different stage of face and/or emotion processing. Scalp event-related potential studies have consistently observed effects of emotional facial expression in similar late time-windows, corresponding to the P300 or Late Positive Potential components (LPP or LPC). These late effects have been generally interpreted as reflecting “cognitive” stages of emotional processing, related in particular to accessing the emotional significance of the facial cue [23], [73] (for reviews see [22], [74]). Altogether, our results thus suggest that the amygdala is involved in multiple stages of emotional face processing.

Gaze is another highly relevant facial cue, particularly in relation with facial emotion perception. The gaze direction of the seen faces modulated amygdala activity between 190 and 350 ms, with greater activity for direct than averted gaze. This effect was more marked in the right amygdala where gaze and emotion interacted, revealing increased activity in response to direct vs. averted gaze only for fearful faces. Several brain imaging studies have provided evidence for amygdala activation by gaze cues [13], [14] and for its involvement in the integration of gaze direction and emotion cues [17]–[19], [63], [75]–[77]. Our data are in line with these prior studies and bring information about the timing of this process. They suggest that the amygdala initially codes the emotional expression and then codes information related to gaze direction; the coding of this information involved sustained activity, emphasizing the importance of gaze and of the integration of gaze and emotional expression cues. Two previous EEG studies reported an interaction between gaze and emotion at 200–300 ms [78], [79]; our results point to the amygdala as a core structure involved in this effect. Our findings also nicely complement and extend recent intracranial data [26], [27].

It is important to mention that the temporal dynamics of amygdala responses to gaze and emotion cues from faces may be bound to the paradigm used. Indeed, in a recent MEG study, we found an early interaction between gaze and emotional expression cues over a right anterior temporo-frontal sensor set [80]. Although there was no source localization in this study, the topographical distribution of the effect suggested that the amygdala was involved, thus subtending an early integration of emotion and gaze cues. However, this study used a very different paradigm, depicting successive and dynamical changes in gaze direction and emotional expressions of pairs of faces, which may have favored the early interaction observed. Furthermore, the sustained interaction between gaze direction and facial expression found here reflected enhanced amygdala activity to fearful faces with direct gaze (relative to fearful faces with averted gaze and to neutral faces with direct gaze). This is in line with the previous fMRI studies that reported greater amygdala activation in response to direct compared to averted gaze fearful faces [63], [75]. However, several other fMRI studies have shown greater amygdala activation in response to averted compared to direct gaze fearful faces [17], [18]. All these studies used different tasks and paradigms, requiring an emotion intensity judgment [18] gender categorization [63], or passive fixation [17], and using only emotional faces [17], [75] or a combination of neutral faces and different types of emotional faces [18], [63]. Altogether, the discrepant results obtained regarding the direction of the interaction between gaze and emotion in the amygdala suggest that task and stimulus parameters may impact greatly on stimulus relevance, hence on the pattern of amygdala responses [7], [72].

We also took into account anxiety as a potential modulator variable of amygdala activity [61]–[63]. Amygdala activity was positively correlated with the subject’s anxiety level. This was observed on all time windows of measurement. This is to our knowledge the first evidence for an anxiety effect on amygdala activity using MEG. Our results are consistent with those of numerous fMRI studies that have demonstrated increased amygdala activation in response to fear-related stimuli associated to anxiety level, both in non-clinical populations [56], [58], [59], [63] and when considering several anxiety-related disorders including generalized anxiety disorder (GAD), post-traumatic stress disorder (PTSD), social anxiety, and phobia [62], [81] (see [62] and [57] for review). This enhanced amygdala activation has been related to a general hypervigilance for emotional – particularly threat-related – stimuli. Indeed, behavioral studies have revealed that anxious individuals show greater attention towards emotional stimuli [82]. Thus, our findings bring further support to the view that amygdala is activated by both endogenous fear-related factors such as anxiety level and exogenous relevant stimuli such as fearful faces.

Moreover, the influence of anxiety level was pervasive: it was observed from the early peak of amygdala activity and it was sustained over the whole time-range of our analysis. This is in line with the hypervigilance hypothesis that postulates an impact of anxiety on the early stage of stimulus processing. It is also consistent with previous EEG and MEG studies that have reported an influence of anxiety at several stages of face processing, both in early (∼100 ms) and late (>200 ms) time-ranges [83]–[94]. Our study expands these prior reports by providing for the first time direct evidence for an early (∼130 ms) influence of anxiety level on the amygdala sources of magnetic activity in response to faces.

It is interesting to note that the effect of anxiety was additive to that of emotion and gaze in the present study. Previous studies have reported that anxiety level modulated amygdala responsiveness to fearful expression and gaze cues [57]–[60], [63]. The reason why such modulation was not observed here is unclear. Some differences may arise from the use of different brain imaging methods. Although it is speculative, it may also be suggested that the lack of amygdala conditional modulation by anxiety might be the counterpart of a greater non-specific reactivity of the amygdala in anxious individuals that could serve to undermine the neural signal-to-noise ratio when processing emotionally relevant environmental cues. In any case, our results are complementary to those of previous studies, suggesting that endogenous and exogenous fear-related anxiety may impact additively on amygdala activity.

The early peak of amygdala activity was concomitant with prominent cortical sources in extrastriate visual cortex. These sources extended into the bilateral occipital regions and the fusiform regions (predominantly in the right hemisphere), and also peaked between 130 and 170 ms. This is consistent with the fact that the early peak of amygdala activity was concurrent with the M170 event-related field pattern at the scalp surface. The fusiform and lateral occipital responses were also modulated by emotion, with greater amplitude of activity in response to fearful than to neutral faces. Although the present study focused on source localization and therefore did not include ERF measurement at the scalp surface, these results are in line with previous studies that have reported emotional modulation of N/M170 [95]–[99] (for a review see [22]). In addition, in line with a recent study of Conty and coll. [76] combining EEG and fMRI, our findings suggest that amygdala activity contributes to the N/M170 recorded at the scalp surface.

The fusiform and lateral occipital responses were also enhanced for fearful relative to neutral faces between 310 and 350 ms. This is in agreement, although pointing to slightly later latencies, with ERP studies that have found early posterior negativity to emotional relative to neutral stimuli, maximum between 200 and 350 ms, with sources in posterior occipito-temporo-parietal regions [100], [101] (for review [102]). The finding of concomitant effects of emotion in both amygdala and extrastriate visual regions is consistent with the view that there is a tight functional coupling between the amygdala and regions of the visual pathway, involving recurrent, dynamic feed-forward and feedback flows of information between these regions [103]–[107]: The visual cortical regions and the amygdala seem to be involved dynamically and in concert in the multiple stages of face processing and emotion perception from faces.

With respect to the effects of gaze and anxiety level, more differentiated effects were obtained in the occipito-temporal regions and in the amygdala. There was not any significant effect of gaze direction in the occipito-temporal clusters, but an interaction between emotion and gaze in the fusiform regions between 190 and 350 ms that reflected greater fusiform activity for fearful compared to neutral faces in the direct gaze condition only. Subject’s anxiety level modulated the activity sustainably in the lateral occipital region only. Altogether it seemed that gaze direction and anxiety had more limited impact on the posterior visual regions than on the amygdala. This is consistent with the central position attributed to the amygdala as a stimulus relevance appraisal system [72].

### Limitations of the Method

Can amygdala activity really be detected and localized from magnetic responses recorded at the scalp surface? The estimation of sources of electromagnetic signal collected outside the head requires solving an inverse problem, and it is therefore a delicate issue. By essence, the inverse problem is an ill-posed problem because of the non-unicity of its solution. In order to constrain its resolution to a limited amount of – and ideally unique – solution, regularization methods have been introduced [108]. Here, we chose to apply a method based on the minimum norm estimate (MNE) [109] that uses a distributed model of the source space with fixed locations. The MNE solution to the inverse problem has the advantage to be unique and to be insensitive to initialization conditions, which are severe limitations of multiple-dipole models. It is widely used to estimate the source distribution with minimal energy (L2-norm) (see [68] for a review). The MNE is however biased towards superficial cortical sources, which is detrimental to the detection of deep sources. This is why we used the depth-weighted version of the classical MNE estimator (wMNE) that corrects for this main bias [110], [111]. In addition, our source model was based on a precise anatomical definition of the source space including neocortex, amygdala, and hippocampus. In a recent assessment study, Attal and Schwartz [112] have quantified the spatial error of subcortical source localization using wMNE and other inverse operators to estimate amygdala and other subcortical sources; their source model was similar to ours (with the addition of the thalamus). According to this simulation study, the spatial localization error in the case of an isolated amygdala activation can be expected to be less than 1cm from the center of gravity of the actual neural currents. This value does not demonstrate a high regional specificity, but it is a promising rating of the quality of our model. Importantly, in the presence of simultaneous cortical and subcortical activations, wMNE was shown to involve the creation of less ghost deeper sources than other inverse operators (dSPM and sLORETA). Moreover, to evaluate the regional specificity of the neural currents estimated from the amygdala, we made complementary analyses of sources in the anterior lateral temporal regions directly between the amygdala and the MEG sensors closest to it, and in the body of the hippocampus near the amygdala region. The results of these analyses are provided in Figure S1. They showed mainly that the time course of responses both in the anterior lateral temporal region and in the hippocampus was notably different from the time course of amygdala responses, with no or limited emotional modulation in the early time range in particular (this emotional modulation was observed in the hippocampus only and could reflect some spreading or cross-talk of amygdala activity). Notably, hippocampal responses were much attenuated in comparison with amygdala responses (see Figure S1). As these structures have similar depth, this is a good hint to a focus of activity in the amygdala.

On a secondary note, it may be reminded here that amygdala responses to faces, and to emotional faces and gaze in particular, have been reported using other brain imaging modalities such as fMRI and intracerebral EEG recording. Taken together with the histological and functional arguments raised in our Introduction, and with the above data from neighboring regions, it forms converging lines supporting the view that we discerned actual amygdala responses.

In sum, we provided a new method for the estimation of amygdala activity from MEG signals using an imaging approach of the inverse modeling problem [50] that included the individual cortical brain surface and the individually segmented amygdala volume [52] as the source space. This approach is complementary to those that have been previously proposed using beamforming or dipole fitting procedures [29], [31], [32], [35], [37], [70], [71] (see [68], [113] for reviews). It confirms the feasibility of the study of amygdala responses with MEG, offering a unique insight in the temporal dynamics of brain responses including deep brain structures as it has been reported for the hippocampus [114]–[116] or the thalamus [36] (see [117] for review).

## Conclusion

This study aimed at examining the neural response of the amygdala to fearful and neutral faces with direct and averted gaze using MEG. We used an original source imaging approach where individually segmented amygdala volumes were included in the source space model. We showed that amygdala is involved in multiple stage of face processing. There was a prominent early peak of amygdala activity between 130 and 170 ms that was enhanced for fearful faces. An effect of emotion was again observed between 310 and 350 ms, suggesting that amygdala was involved in different stages of the fearful face appraisal. Moreover, amygdala activity was modulated by gaze direction between 190 and 350 ms, with greater response to direct gaze faces and a more marked effect of gaze for the fearful faces in the right amydgala. Altogether, our results underline the role of the amydgala in the processing of social cues from faces. They promote MEG source imaging techniques as fruitful tools for the non invasive study of the temporal dynamics of neural responses from cortical and subcortical brain regions in healthy subjects.

## Supporting Information

Figure S1 Time course of the neural responses to fearful and neutral faces with direct and averted gaze in the lateral anterior temporal region (in A) and in the body of the hippocampus (in B). A) The time courses of the cortical source activity averaged across all subjects over the right and left lateral anterior temporal clusters respectively (displayed in red on lateral views of the template brain, in small insets) are presented. These time courses were notably different from the time course of amygdala responses, lacking the prominent peak of activation obtained in the amygdala between 130 and 170 ms; furthermore, the mean amplitude of anterior temporal activities between 130 and 170 ms was not modulated by emotional expression (F<1); in the later time range, (190–350 ms) there was only a very localised effect of gaze between 230 and 270 ms in the right hemisphere and for fearful faces only (F(1, 13) = 5.90, p<.04; the interactions between gaze, time window, and emotion, and between gaze, time window, emotion, and hemisphere were significant; F(3,39) = 4.59, ε_GG_ = 0.88, p<.01 and F(3,39) = 3.95, ε_GG_ = 0.69, p<.02 respectively). B) The time courses of hippocampus body sources averaged across all subjects over the right and left hippocampus body clusters respectively (defined with kmeans in each individual, as displayed in red on a typical left hippocampus mesh, in the small inset) is presented. These time courses showed a peak activity between 130 and 170 ms that was of markedly attenuated amplitude in comparison with the amygdala early peak, and weak later sustained response. The mean amplitude of hippocampus activity between 130 and 170 ms yielded a significant effect of emotion (F(1, 13) = 8.32, p<.02) that could reflect some spreading or cross-talk of amygdala activity. In contrast with the results obtained for the amygdala, there was not any effect of gaze on the mean amplitude of hippocampus response between 190 and 350 ms (F(1, 13)<3, p>.1). The areas shaded in grey represents the time windows where mean amplitude measurements were performed (see main text).
(TIF)
