# Correction: MEG Evidence for Dynamic Amygdala Modulations by Gaze and Facial Emotions

**DOI:** 10.1371/annotation/0613c203-5f8a-4aec-b15d-0324bc5788f8

**Published:** 2013-10-01

**Authors:** Thibaud Dumas, Stéphanie Dubal, Yohan Attal, Marie Chupin, Roland Jouvent, Shasha Morel, Nathalie George

There was an error in Reference 117. The correct version of this Reference is available below.

Attal Y, Maess B, Friederici A, David O (2012) Head models and dynamic causal modeling of subcortical activity using magnetoencephalographic/electroencephalo​graphicdata. Rev Neurosci 23: 85–95. doi: 10.1515/rns.2011.056. 

