# Supplementary figures and images for: MEG Evidence for Dynamic Amygdala Modulations by Gaze and Facial Emotions
Source: PLoS One. 2013 Sep 10;8(9):e74145. doi: 10.1371/journal.pone.0074145 (PMC3769395; doi:10.1371/journal.pone.0074145)

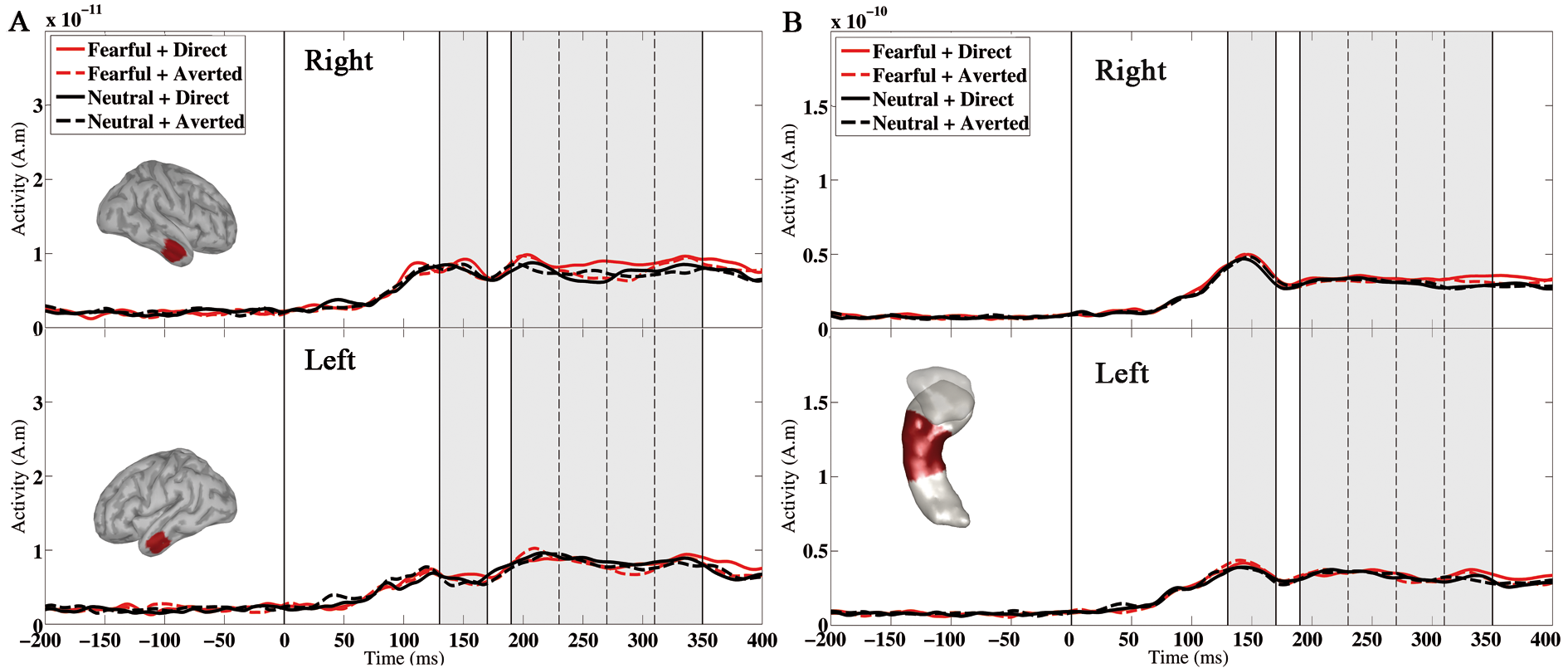

Supplement: Figure S1 — Time course of the neural responses to fearful and neutral faces with direct and averted gaze in the lateral anterior temporal region (in A) and in the body of the hippocampus (in B). A) The time courses of the cortical source activity averaged across all subjects over the right and left lateral anterior temporal clusters respectively (displayed in red on lateral views of the template brain, in small insets) are presented. These time courses were notably different from the time course of amygdala responses, lacking the prominent peak of activation obtained in the amygdala between 130 and 170 ms; furthermore, the mean amplitude of anterior temporal activities between 130 and 170 ms was not modulated by emotional expression (F<1); in the later time range, (190–350 ms) there was only a very localised effect of gaze between 230 and 270 ms in the right hemisphere and for fearful faces only (F(1, 13) = 5.90, p<.04; the interactions between gaze, time window, and emotion, and between gaze, time window, emotion, and hemisphere were significant; F(3,39) = 4.59, εGG = 0.88, p<.01 and F(3,39) = 3.95, εGG = 0.69, p<.02 respectively). B) The time courses of hippocampus body sources averaged across all subjects over the right and left hippocampus body clusters respectively (defined with kmeans in each individual, as displayed in red on a typical left hippocampus mesh, in the small inset) is presented. These time courses showed a peak activity between 130 and 170 ms that was of markedly attenuated amplitude in comparison with the amygdala early peak, and weak later sustained response. The mean amplitude of hippocampus activity between 130 and 170 ms yielded a significant effect of emotion (F(1, 13) = 8.32, p<.02) that could reflect some spreading or cross-talk of amygdala activity. In contrast with the results obtained for the amygdala, there was not any effect of gaze on the mean amplitude of hippocampus response between 190 and 350 ms (F(1, 13)<3, p>.1). The areas shaded in grey [file pone.0074145.s001.tif]
